# Prognostic significance of MYCN related genes in pediatric neuroblastoma: a study based on TARGET and GEO datasets

**DOI:** 10.1186/s12887-020-02219-1

**Published:** 2020-06-27

**Authors:** Haiwei Wang, Xinrui Wang, Liangpu Xu, Ji Zhang, Hua Cao

**Affiliations:** 1 Medical Research Center, Fujian Maternity and Child Health Hospital, Affiliated Hospital of Fujian Medical University, Fuzhou, Fujian China; 2 State Key Laboratory for Medical Genomics, Shanghai Institute of Hematology, Rui-Jin Hospital Affiliated to School of Medicine, Shanghai Jiao Tong University, Shanghai, China

**Keywords:** Pediatric neuroblastoma, MYCN, E2F1, EIF4G1, Ribosome signaling pathway, TARGET, GEO

## Abstract

**Background:**

Neuroblastoma patients with MYCN amplification are associated with poor prognosis. However, the prognostic relevance of MYCN associated genes in neuroblastoma is unclear.

**Methods:**

The expression profiles of MYCN associated genes were identified from Therapeutically Applicable Research to Generate Effective Treatments (TARGET) and Gene Expression Omnibus (GEO) datasets. Enriched transcription factors and signaling pathways were determined using gene set enrichment analysis (GSEA). Kaplan-Meier plotter was used to identify the prognostic relevance of MYCN associated genes. Multivariate cox regression and Spearman’s correlation were used to determine the correlation coefficients of MYCN associated genes.

**Results:**

In TARGET and GSE85047 datasets, neuroblastoma patients with MYCN amplification were associated with worse prognosis. Transcription factor MYC was positively associated with MYCN amplification in GSEA assay. We identified 13 MYC target genes which were increased in neuroblastoma patients with MYCN amplification in TARGET, GSE19274 and GSE85047 datasets. Moreover, six out of the 13 MYC target genes ARMC6, DCTPP1, EIF4G1, ELOVL6, FBL and PRMT1 were associated with adverse prognosis in TARGET and GSE85047 datasets. Transcription factor E2F1 was up-regulated by MYCN amplification and associated with the poor prognosis of neuroblastoma. Furthermore, RPS19 in ribosome signaling pathway was also associated with MYCN amplification and correlated with the poor prognosis of neuroblastoma. At last, we showed that most of MYCN target genes were correlated with each other. However, EIF4G1 was an independent prognostic marker. And the prognostic effects of the combination of MYCN amplification and EIF4G1 expression were more significant than MYCN or EIF4G1 alone.

**Conclusions:**

MYCN target genes ARMC6, DCTPP1, EIF4G1, ELOVL6, FBL, PRMT1, E2F1 and RPS19 had significant prognostic effects in pediatric neuroblastoma. And neuroblastoma patients without MYCN amplification and low EIF4G1 expression had best prognosis.

## Background

Neuroblastoma is a common pediatric solid tumor derived from the sympathetic nervous system and a major cause of pediatric cancer related mortality [1, 2]. Neuroblastoma is highly heterogeneous and the prognosis of neuroblastoma is highly variable. Some neuroblastoma rapidly regresses with standard treatment, while, other neuroblastoma progresses despite the extensive treatment [3]. Age, tumor stage [4], tumor ploidy [5], Aurora kinase A expression [6], hypoxia gene signature [7] and RAS and/or TP53 mutations [8] are all used for the prediction of the clinical outcomes of neuroblastoma. However, new prognostic markers are still needed.

MYCN amplification represents the strongest independent adverse prognostic factor [9]. MYCN belongs to the MYC transcription factor family and is amplified in approximate 25% of neuroblastoma patients [10]. According to the International Neuroblastoma Risk Group, neuroblastoma patients are classified into low, intermediate or high risk subgroups based on MYCN amplification status [11]. Patients with MYCN amplification are associated with high risk and worse prognostic outcome [12]. Except MYCN amplification, MYCN protein expression [13], MYCN target gene CD44 [14] and MYCN signature [15] are used to predict the outcome of neuroblastoma. As a master transcription factor, MYCN controls the transcriptional activity of multiple target genes [16]. However, the globe MYCN amplification regulated genes and their predictive relevance in neuroblastoma are unclear.

In the present study, using published TARGET [17] and GEO datasets, we comprehensively analyzed the differentially expressed genes in neuroblastoma patients with or without MYCN amplification, and identified the critical signaling pathways and transcription factors involving MYCN regulation. We also analyzed the prognostic effects of MYCN target genes in pediatric neuroblastoma. Overall, we found eight MYCN target genes ARMC6, DCTPP1, EIF4G1, ELOVL6, FBL, PRMT1, E2F1 and RPS19 which had significant prognostic effects in pediatric neuroblastoma patients.

## Methods

### Data collection

The clinical and expression datasets of neuroblastoma patients were downloaded from the TARGET project, launched by national cancer institute (https://ocg.cancer.gov/). Expression series matrix of neuroblastoma tissues was also downloaded from GEO website (https://www.ncbi.nlm.nih.gov/geo/), including GSE19274, GSE73517, GSE49710 and GSE85047 datasets.

### Prognostic effects of MYCN amplification

Clinical data of neuroblastoma patients deposited in TARGET and GSE85047 was used to determine the different overall survival of neuroblastoma patients with or without MYCN amplification. Log-rank test was used to determine the *P* values.

### GEO data processing

The gene expression matrix was annotated with corresponding platform. The expression values were processed using R software (R version 3.5.0) “plyr” package (version 1.8.5; https://cran.r-project.org/web/packages/plyr/index.html).

### Gene set enrichment analysis (GSEA)

Signaling pathway and transcription factor enrichment was performed using GSEA 2.0 software (http://www.broad.mit.edu/gsea/index.html). Statistical significance *P*-values were determined by 1000 gene set permutations.

### Heatmap presentation

Heatmaps were generated by “pheatmap” package (version 1.0.12; https://cran.r-project.org/web/packages/pheatmap/ index.html) in R software. The clustering scale was determined by “average” method. The clustering distance was determined by the ‘correlation’ method.

### Prognostic effects of MYCN target genes

‘Survival’ package (version 3.1–8; https://cran.r-project.org/web/packages/survival/ index.html) in R software was used to determine the clinical influence of MYCN target genes. The neuroblastoma patients were divided into two subgroups based on the mean expression levels of MYCN target genes. Log-rank test was used to test the different clinical outcomes in neuroblastoma patients with high expression levels and low expression levels of MYCN target genes.

### Multivariate cox regression

R software ‘survival’ package (version 3.1–8) was used for multivariate cox regression analysis. Log-rank test was used to calculate the *P* values based on ‘coxph’ method.

### Correlation plots of MYCN target genes

Correlation plots of MYCN target genes were created using the ‘corrplot’ package (version 0.84; https://cran.r-project.org/web/packages/corrplot/index.html) in R software. The correlation coefficients were determined by Spearman’s correlation test.

### Statistical analysis

The box plots were generated from GraphPad Prism software (version 5.0; https://www.graphpad.com/). Statistical analysis was performed using the Student’s t test using R software. *P* value less than 0.05 was chosen to be statistically significant difference.

## Results

### Prognostic significance of MYCN amplification in patients with neuroblastoma

**Fig. 1:**
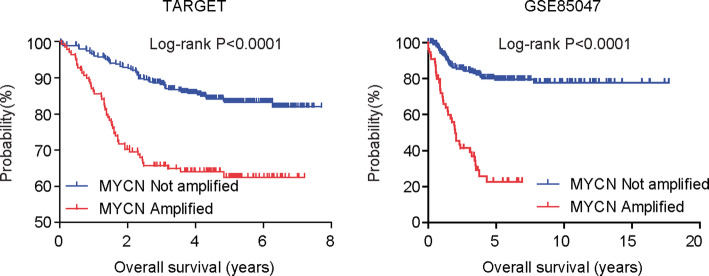
Prognostic significance of MYCN amplification in patients with neuroblastoma. Kaplan-Meier plots showed the prognostic relevance of MYCN amplification in patients with neuroblastoma in both TARGET and GSE85047 datasets. Different overall survival between patients with (red) or without (blue) MYCN amplification was determined by log-rank test

Using the clinical data deposited in TARGET dataset, we analyzed the prognosis of MYCN amplification in patients with neuroblastoma. Consistent with the previous report that MYCN amplification was associated with poor outcome [12], neuroblastoma patients with MYCN amplification had worse prognosis than patients without MYCN amplification in TARGET dataset (Fig. 1). The prognostic effects of MYCN amplification were further validated in GSE85047 dataset. Neuroblastoma patients with MYCN amplification also demonstrated worse clinical outcomes in GSE85047 dataset (Fig. 1).

### Identification of MYCN target genes in patients with neuroblastoma

**Fig. 2:**
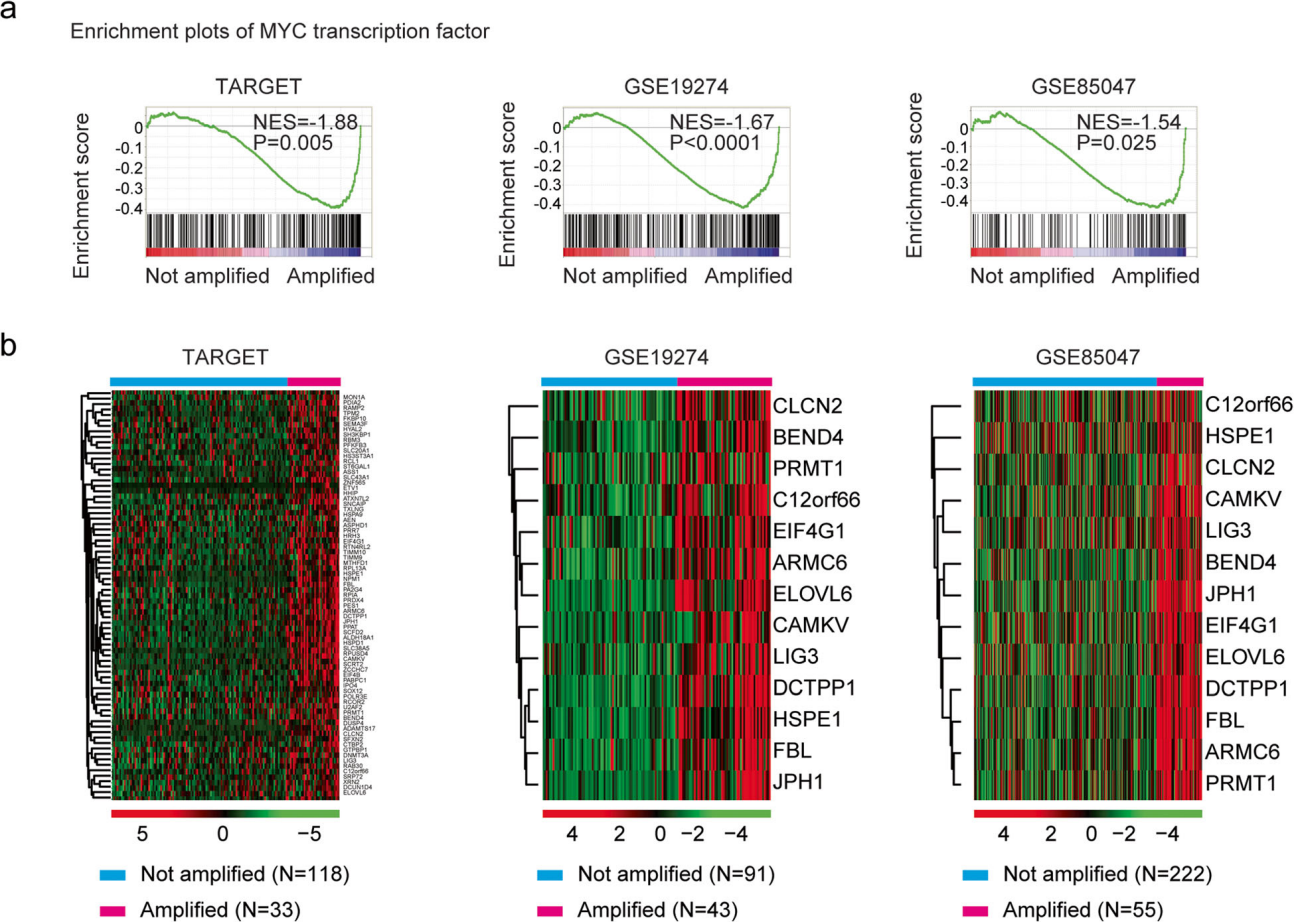
Identification of MYCN target genes in patients with neuroblastoma. **a** Enrichment plots of transcription factor MYC in TARGET, GSE19274 and GSE85047 datasets. Enrichment of normalized enrichment score (NES) and *P*-values were presented. **b** Heatmaps demonstrated the expression profiles of MYC target genes in MYCN amplified and not amplified neuroblastoma tissues in TARGET, GSE19274 and GSE85047 datasets. Genes up-regulated (red), down-regulated (blue) and moderately regulated (black) were delineated

To reveal the functional relevance of MYCN regulated genes in neuroblastoma, we performed transcription factor enrichment analysis through GSEA assay. We found that MYC transcription factor was positively correlated with the MYCN amplification in neuroblastoma in TARGET dataset (Fig. 2a). Transcription factor MYC regulates multiple target genes. In the GSEA assay, we identified 75 MYC target genes which were up-regulated in MYCN amplified neuroblastoma tissues in TARGET dataset (Fig. 2b).

The positive enrichment of MYC transcription factor in MYCN amplified neuroblastoma tissues was also observed in GSE19274 and GSE85047 datasets (Fig. 2a). We further validated 13 out of 75 MYC target genes ARMC6, BEND4, C12orf66, CAMKV, CLCN2, DCTPP1, EIF4G1, ELOVL6, FBL, HSPE1, JPH1, LIG3 and PRMT1 which were up-regulated in MYCN amplified neuroblastoma tissues in GSE19274 and GSE85047 datasets (Fig. 2b).

### Prognostic significance of MYCN target genes in patients with neuroblastoma: analysis from TARGET dataset

**Fig. 3:**
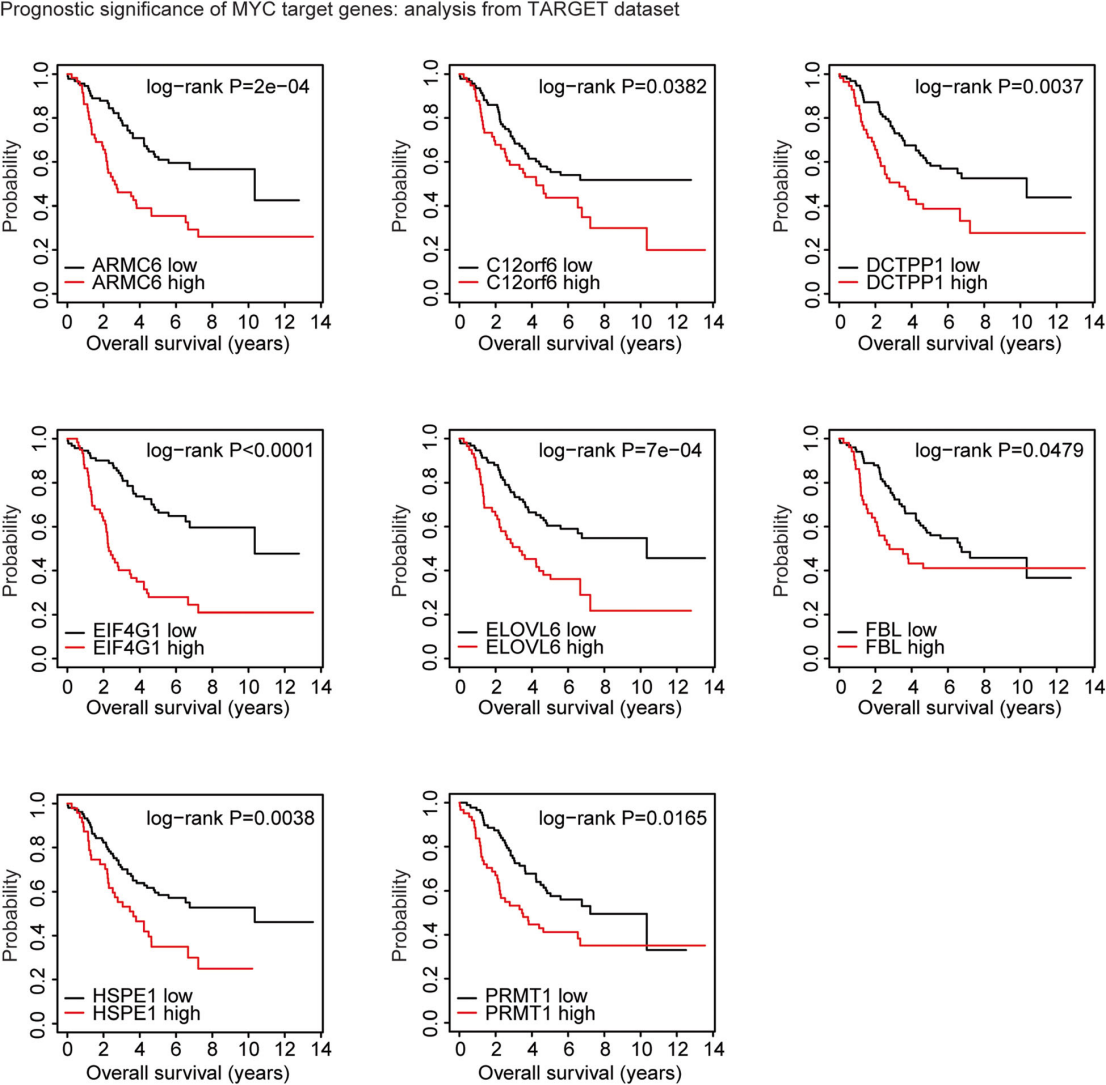
Prognostic significance of MYCN target genes in patients with neuroblastoma: analysis from TARGET dataset. The Kaplan-Meier plots demonstrated the prognostic effects of MYCN target genes ARMC6, C12orf6, DCTPP1, EIF4G1, ELOVL6, FBL, HSPE1 and PRMT1 in patients with neuroblastoma using TARGET dataset. Patients were divided into two clusters based on the mean expression levels of the MYCN target genes. The log-rank test was used to determine the overall survival *P*-values

Since MYCN amplification was associated with poor outcome in neuroblastoma, we next assessed the prognostic effects of the 13 MYC target genes which were up-regulated in MYCN amplified neuroblastoma. We found that, high expression levels of MYCN target genes ARMC6, C12orf6, DCTPP1, EIF4G1, ELOVL6, FBL, HSPE1 and PRMT1 were associated with worse prognosis in TARGET dataset (Fig. 3). However, other MYCN target genes BEND4, CAMKV, CLCN2, JPH1 and LIG3 demonstrated no prognostic significance.

### Prognostic significance of MYCN target genes in patients with neuroblastoma: analysis from GSE85047 dataset

**Fig. 4:**
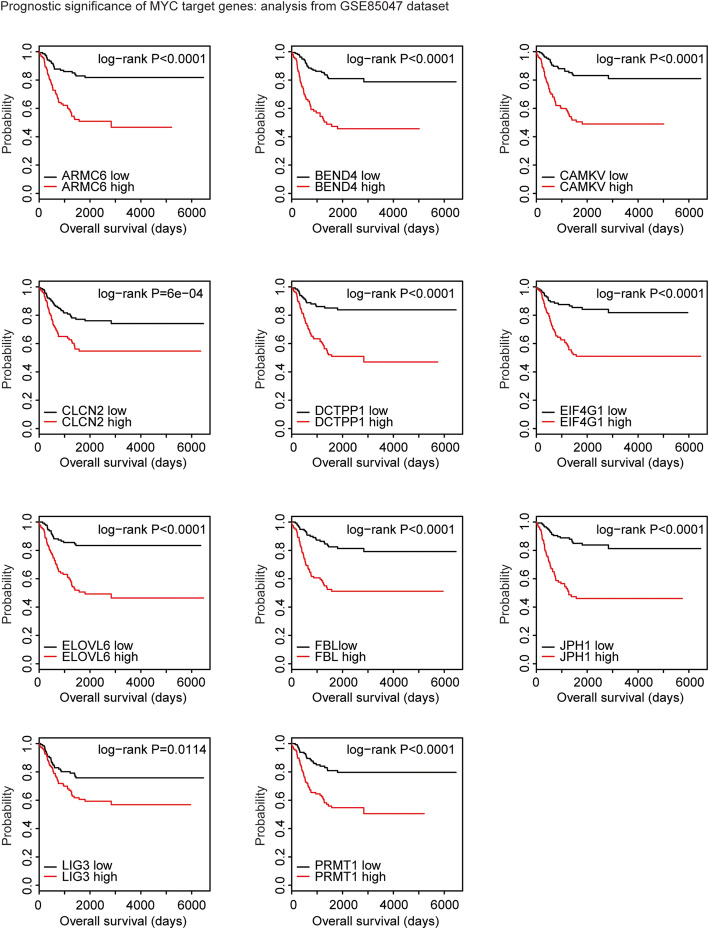
Prognostic significance of MYCN target genes in patients with neuroblastoma: analysis from GSE85047 dataset. The Kaplan-Meier plots demonstrated the prognostic effects of MYCN target genes ARMC6, BEND4, CAMKV, CLCN2, DCTPP1, EIF4G1, ELOVL6, FBL, JPH1, LIG3 and PRMT1 in patients with neuroblastoma using GSE85047 dataset

The prognostic effects of 13 MYC target genes were further validated in GSE85047 dataset. 11 out of the 13 MYC target genes, ARMC6, BEND4, CAMKV, CLCN2, DCTPP1, EIF4G1, ELOVL6, FBL, JPH1, LIG3 and PRMT1 were all correlated with the worse prognosis in patients with neuroblastoma in GSE85047 dataset. Patients with high expression levels of those genes had low overall survival time (Fig. 4). Interestingly, six genes ARMC6, DCTPP1, EIF4G1, ELOVL6, FBL and PRMT1 were associated with the clinical overall survival of neuroblastoma in both TARGET and GSE85047 datasets.

### E2F1 is regulated by MYCN amplification and associated with the prognosis of neuroblastoma

**Fig. 5:**
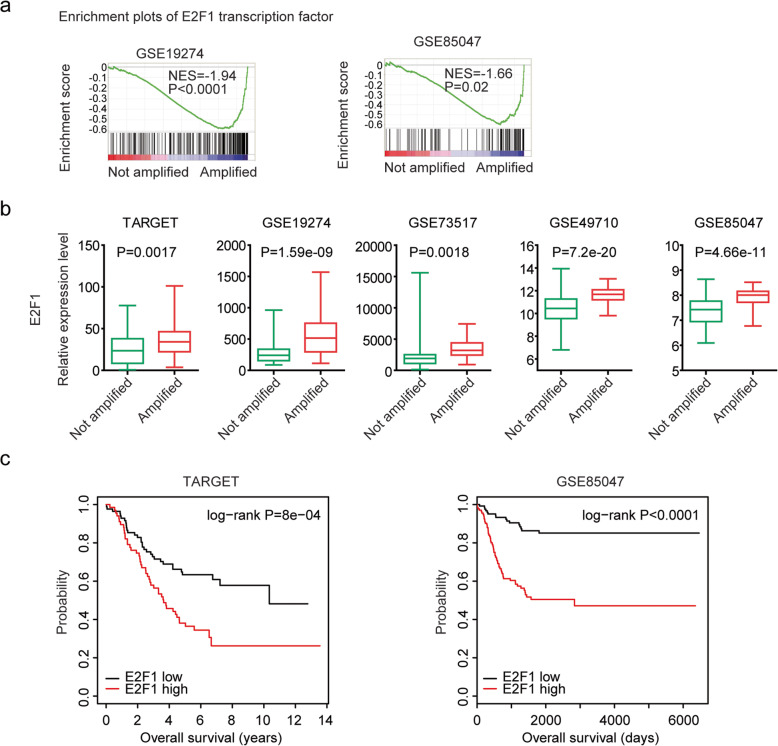
E2F1 is regulated by MYCN amplification and associated with the prognosis of neuroblastoma. **a** Enrichment plots of transcription factor E2F1 in GSE19274 and GSE85047 datasets. **b** Box plots showed the relative E2F1 expression levels in neuroblastoma patients with (red) or without (green) MYCN amplification in TARGET, GSE19274, GSE73517, GSE49710 and GSE85047 datasets. *P* values were performed using Student’s t test. **c** The Kaplan-Meier plots demonstrated the prognostic effects of E2F1 in patients with neuroblastoma in TARGET and GSE85047 datasets

Except MYC, transcription factor E2F1 was also positively enriched in MYCN amplified neuroblastoma tissues in GSE19274 and GSE85047 datasets (Fig. 5a). The high expression levels of E2F1 were observed in neuroblastoma patients with MYCN amplification in TARGET, GSE19274, GSE73517, GSE49710 and GSE85047 datasets (Fig. 5b). And high expression of E2F1 was associated with poor prognosis in patients with neuroblastoma in TARGET and GSE85047 datasets (Fig. 5c).

### RPS19 is regulated by MYCN amplification and associated with the prognosis of neuroblastoma

**Fig. 6:**
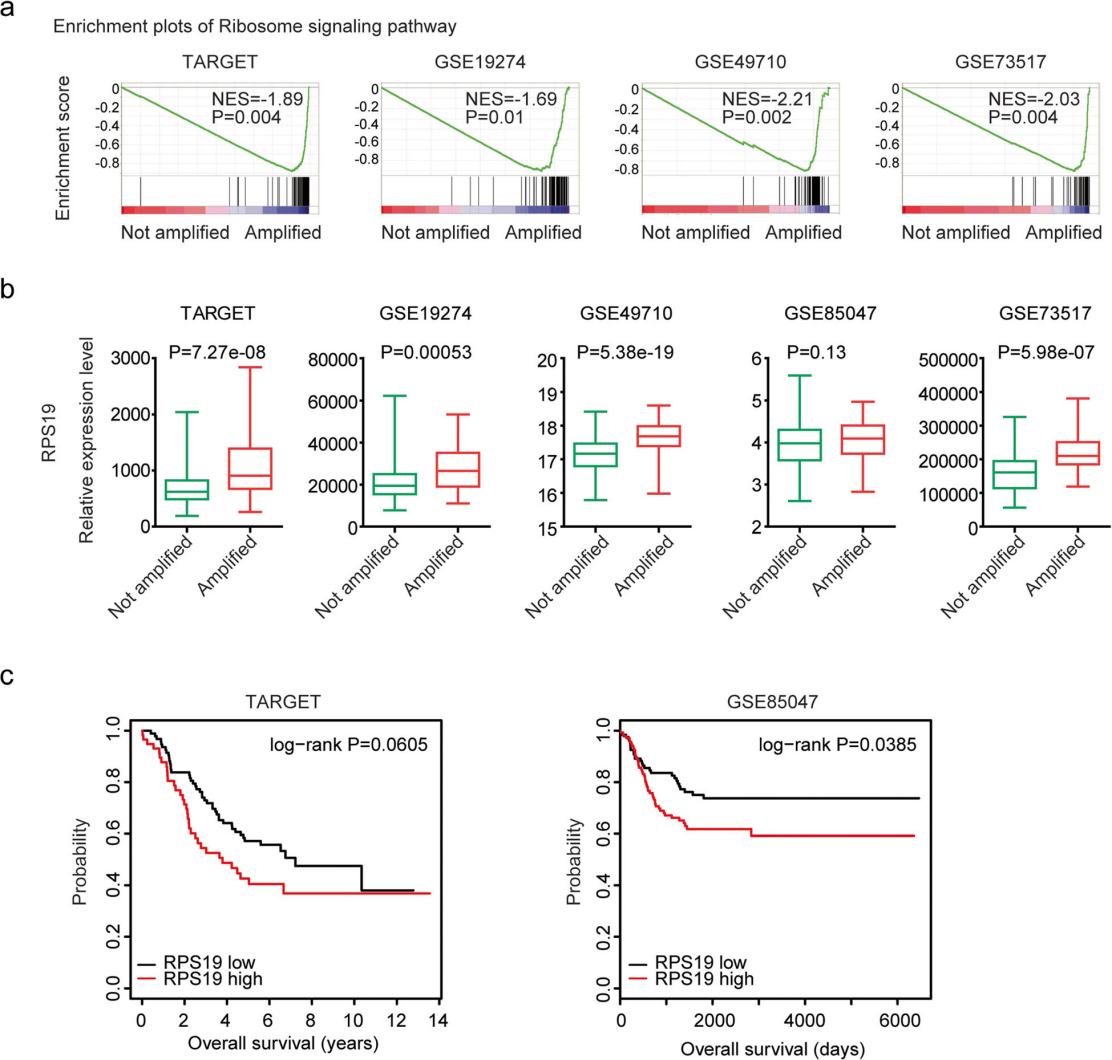
RPS19 is regulated by MYCN amplification and associated with the prognosis of neuroblastoma. **a** Enrichment plots of ribosome signaling pathway in TARGET, GSE19274, GSE49710 and GSE85047 datasets. **b** Box plots showed the relative RPS19 expression levels in neuroblastoma patients with (red) or without (green) MYCN amplification in TARGET, GSE19274, GSE49710, GSE73517 and GSE85047 datasets. **c** The Kaplan-Meier plots demonstrated the prognostic effects of RPS19 in patients with neuroblastoma in TARGET and GSE85047 datasets

We also identified the functional signaling pathways which were associated with MYCN amplification in neuroblastoma. We found that ribosome signaling pathway represented the most frequently enriched signaling pathway in TARGET, GSE19274, GSE49710 and GSE85047 datasets (Fig. 6a). Most genes in ribosome signaling pathway were up-regulated in MYCN amplified neuroblastoma patients, as demonstrated the RPS19 expression levels in TARGET, GSE19274, GSE49710, GSE73517 and GSE85047 datasets (Fig. 6b). However, most genes in ribosome signaling pathway were not associated with the prognosis of neuroblastoma. Only, RPS19 demonstrated poor prognostic effects in patients with neuroblastoma in TARGET and GSE85047 datasets (Fig. 6c).

### Correlation of MYCN target genes in patients with neuroblastoma

**Fig. 7:**
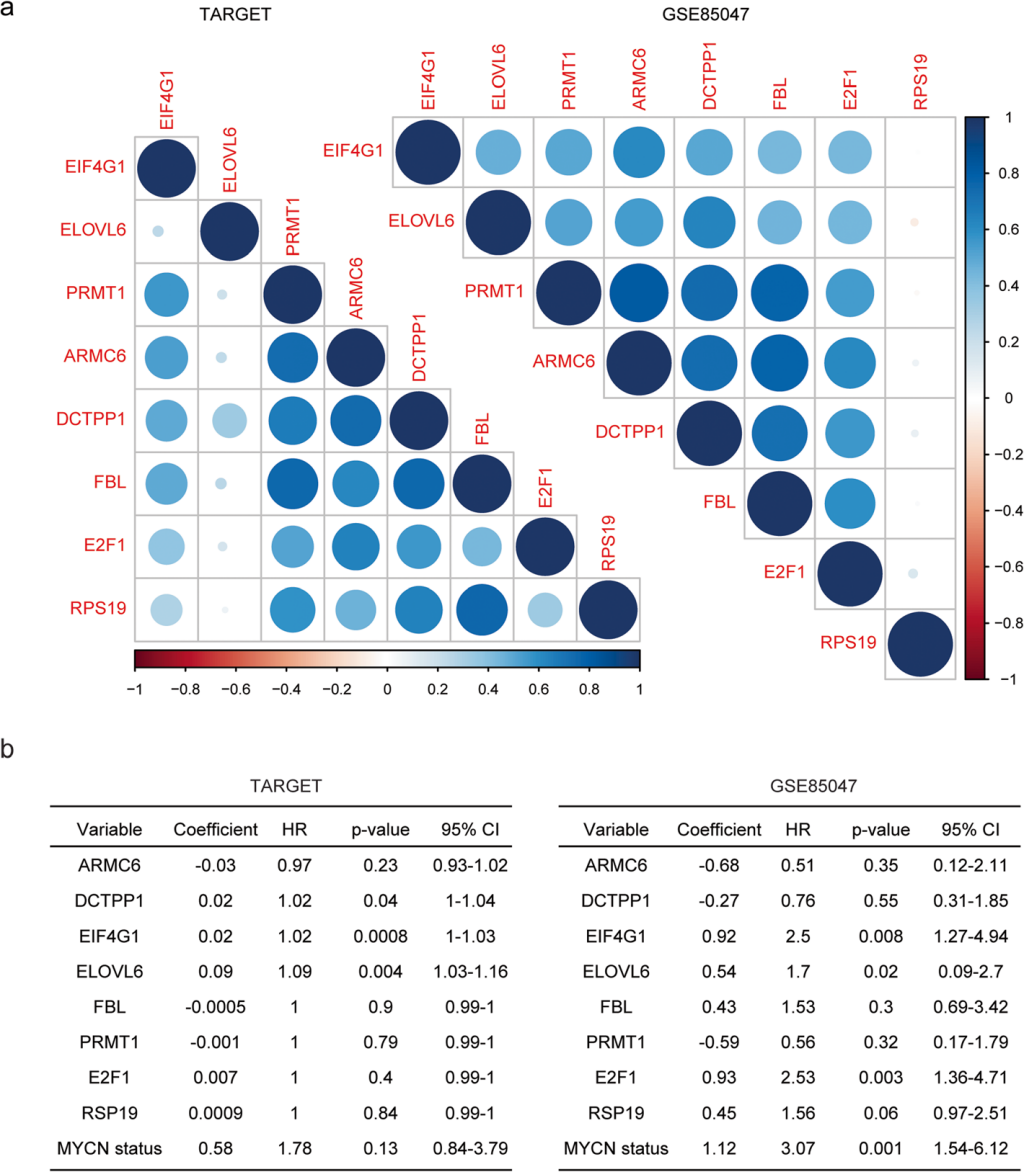
Correlation of MYCN target genes in patients with neuroblastoma. **a** Corrplots demonstrated the correlation of MYCN target genes ARMC6, DCTPP1, EIF4G1, ELOVL6, FBL, PRMT1, E2F1 and RPS19 in TARGET and GSE85047 datasets. The color and the size of the circle represented the correlation coefficients. **b** Multivariate cox regression was used to determine the association of MYCN target genes in neuroblastoma patients in TARGET and GSE85047 datasets

So far, we identified eight MYCN target genes ARMC6, DCTPP1, EIF4G1, ELOVL6, FBL, PRMT1, E2F1 and RPS19 which were up-regulated in MYCN amplified neuroblastoma patients and associated with worse prognosis of neuroblastoma in TARGET and GSE85047 datasets. Next, we determined the correlation of those genes based on their expression levels. ARMC6, DCTPP1, EIF4G1 FBL, PRMT1, E2F1 and RPS19 were highly associated with each other in TARGET dataset (Fig. 7a). However, ELOVL6 was not correlated with other MYCN target genes (Fig. 7a). In GSE85047 dataset, MYCN target genes were correlated with each other except RPS19 (Fig. 7a).

Furthermore, we used multivariate cox regression to determine the association of MYCN target genes in neuroblastoma patients in TARGET and GSE85047 datasets. We found that DCTPP1, EIF4G1 and ELOVL6 were independent prognostic markers in TARGET dataset (Fig. 7b). In GSE85047 dataset, EIF4G1, ELOVL6 and E2F1 were independent prognostic markers (Fig. 7b). Moreover, MYCN amplification was also an independent prognostic marker in patients with neuroblastoma in GSE85047 dataset (Fig. 7b).

### Combined prognostic significance of MYCN amplification and EIF4G1 expression in patients with neuroblastoma

**Fig. 8:**
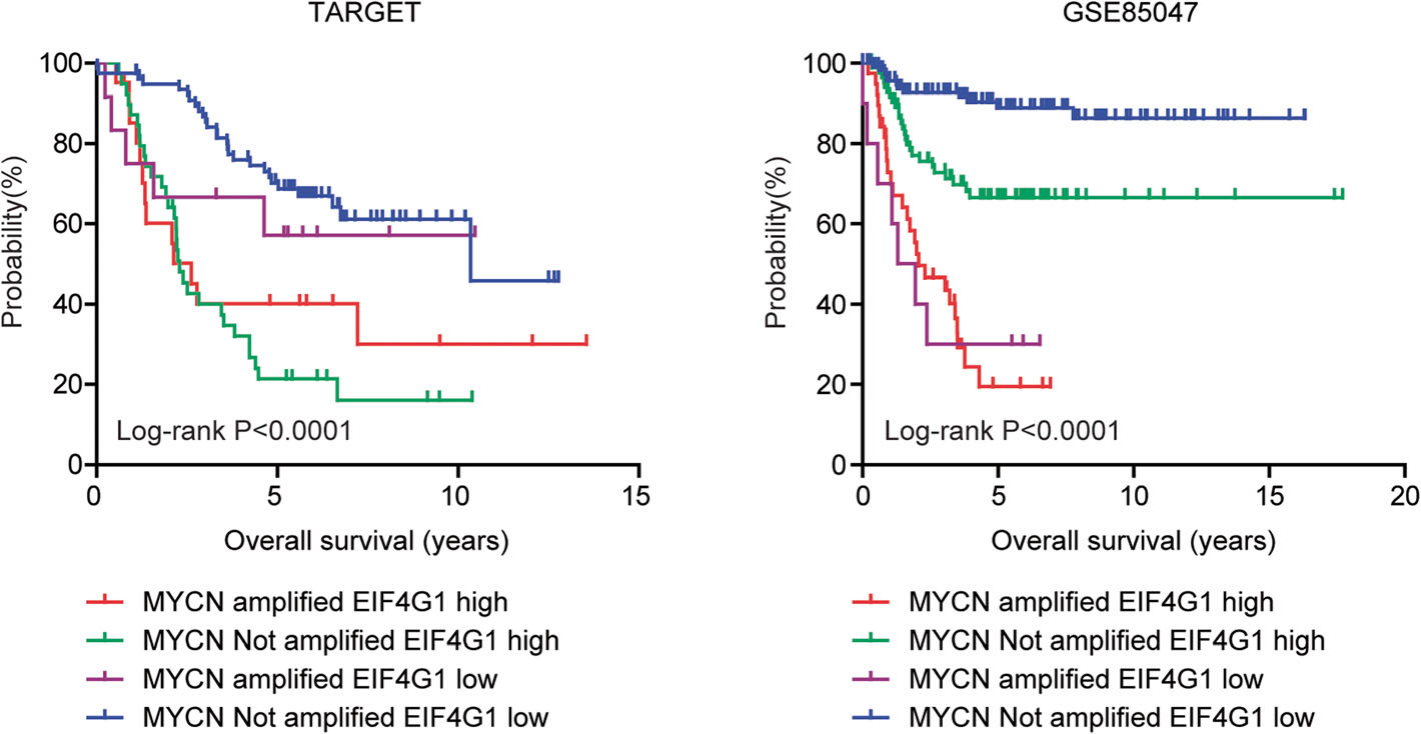
Combined prognostic significance of MYCN amplification and EIF4G1 expression in patients with neuroblastoma. The Kaplan-Meier plots demonstrated the different overall survival of neuroblastoma patients with different MYCN amplification status and EIF4G1 expression levels in TARGET and GSE85047 datasets. Log-rank test was used to determine the *P* values

In both TARGET and GSE85047 datasets, EIF4G1 was a strong independent prognostic marker. So, we tested the combinational prognostic effects of EIF4G1 expression and MYCN amplification in patients with neuroblastoma. Neuroblastoma patients were divided into four sub-groups based on MYCN status and EIF4G1 mean expression level in TARGET and GSE85047 datasets. Neuroblastoma patients with MYCN amplification and high EIF4G1 expression demonstrated worse clinical outcomes in TARGET dataset (Fig. 8). In GSE85047 dataset, neuroblastoma patients without MYCN amplification were divided into EIF4G1 highly expressed group and EIF4G1 lowly expressed group. We found that neuroblastoma patients without MYCN amplification and with low EIF4G1 expression had best prognosis (Fig. 8).

## Discussion

As the drive alteration in neuroblastoma, prognostic signature associated with MYCN amplification was previously identified. Using MYC target database, results showed that MYC pathway activity was associated with the poor outcome of neuroblastoma [18]. Using neuroblastoma SHEP-21 N cell line with ectopic expression of MYCN [19] or shRNA mediated silencing of MYCN [15], the functional MYCN signatures were revealed. Cohort studies demonstrated the prognostic significance of the MYCN signatures derived from SHEP-21 N cell line [20]. However, the overlaps between different prognostic signatures were limited and results from MYCN silencing in neuroblastoma IMR32 cell line demonstrated a completely different prognostic signature [15]. Those observations highlighted the importance of comprehensive and integrated analysis of different cohort of neuroblastoma patients in order to obtain a more precise MYCN related prognostic signature.

So, in the present study, through integrated analysis of TARGET and GEO datasets, we identified eight MYCN amplification associated genes ARMC6, DCTPP1, EIF4G1, ELOVL6, FBL, PRMT1, E2F1 and RPS19 which had significant prognostic effects in pediatric neuroblastoma patients. Previous results showed that PRMT1 regulated MYCN expression in neuroblastoma [21], and down-regulation of PRMT1 induced the senescence of non-MYCN amplified neuroblastoma cells [22]. E2F1 was a therapeutic target in neuroblastoma. Inhibition of E2F1 suppressed neuroblastoma progression [23]. Inhibition of ribosome singling pathway was also a promising strategy in neuroblastoma treatment [24]. However, the functions and prognosis of ARMC6, DCTPP1, EIF4G1, ELOVL6 and FBL in neuroblastoma were not reported.

The purpose of this study was to determine the transcription factors and signaling pathways associated with MYCN amplification and identify the prognostic relevance of MYCN associated genes in neuroblastoma. The present study suggested new prognostic markers of ARMC6, DCTPP1, EIF4G1, ELOVL6 and FBL in neuroblastoma and highlighted the combinational prognostic significance of MYCN amplification and EIF4G1 expression in patients with neuroblastoma. However, the results were derived from published TARGET and GEO datasets and lack of further validation in neuroblastoma patients. Therefore, functions and prognosis of ARMC6, DCTPP1, EIF4G1, ELOVL6 and FBL in neuroblastoma should be further studied.

## Conclusions

MYC, E2F1 transcription factors and ribosome signaling pathway were significantly enriched in neuroblastoma patients with MYCN amplification. MYCN target genes ARMC6, DCTPP1, EIF4G1, ELOVL6, FBL, PRMT1, E2F1 and RPS19 which had significant prognostic effects in pediatric neuroblastoma patients. Neuroblastoma patients without MYCN amplification and low EIF4G1 expression had best prognosis.

## Data Availability

The datasets used and analyzed during the current study are available from the corresponding author on reasonable request.

## Abbreviations

TARGET: Therapeutically Applicable Research to Generate Effective Treatments
GEO: Gene Expression Omnibus;
GSEA: Gene set enrichment analysis
NES: Normalized enrichment score
